# Evaluating Scoliosis Severity Based on Posturographic X-ray Images Using a Contrastive Language–Image Pretraining Model

**DOI:** 10.3390/diagnostics13132142

**Published:** 2023-06-22

**Authors:** Artur Fabijan, Robert Fabijan, Agnieszka Zawadzka-Fabijan, Emilia Nowosławska, Krzysztof Zakrzewski, Bartosz Polis

**Affiliations:** 1 Department of Neurosurgery, Polish-Mother’s Memorial Hospital Research Institute, 93-338 Lodz, Poland; emilia.nowoslawska@iczmp.edu.pl (E.N.); krzysztof.zakrzewski@iczmp.edu.pl (K.Z.); jezza@post.pl (B.P.); 2 Independent Researcher, Luton, LU2 0GS, UK; robert.f.fabijan@gmail.com; 3 Department of Rehabilitation Medicine, Medical University of Lodz, 90-419 Lodz, Poland; agnieszka.zawadzka@umed.lodz.pl

**Keywords:** artificial intelligence, scoliosis, CLIP, OpenAI, ChatGPT, contrastive language–image pretraining, open-source artificial intelligence models

## Abstract

Assessing severe scoliosis requires the analysis of posturographic X-ray images. One way to analyse these images may involve the use of open-source artificial intelligence models (OSAIMs), such as the contrastive language–image pretraining (CLIP) system, which was designed to combine images with text. This study aims to determine whether the CLIP model can recognise visible severe scoliosis in posturographic X-ray images. This study used 23 posturographic images of patients diagnosed with severe scoliosis that were evaluated by two independent neurosurgery specialists. Subsequently, the X-ray images were input into the CLIP system, where they were subjected to a series of questions with varying levels of difficulty and comprehension. The predictions obtained using the CLIP models in the form of probabilities ranging from 0 to 1 were compared with the actual data. To evaluate the quality of image recognition, true positives, false negatives, and sensitivity were determined. The results of this study show that the CLIP system can perform a basic assessment of X-ray images showing visible severe scoliosis with a high level of sensitivity. It can be assumed that, in the future, OSAIMs dedicated to image analysis may become commonly used to assess X-ray images, including those of scoliosis.

## 1. Introduction

Open-source artificial intelligence models (OSAIMs) mostly comprise free, publicly available tools that can be used in various fields, such as computer science and medicine [1,2]. One type of program belonging to OSAIMs includes systems created for image analysis; however, these programs are not currently dedicated to the evaluation of medical images, including X-rays.

In recent months, global interest in OSAIMs has increased. This surge in worldwide attention is partly due to the release of an advanced language model called ChatGPT, produced by the company OpenAI. One of OpenAI’s products is contrastive language–image pretraining (CLIP), which integrates both natural language and images. This state-of-the-art model is capable of a wide range of tasks, including zero-shot image classification, image captioning, and visual question answering [3]. CLIP achieves its remarkable results by training on a diverse set of images as well as their textual descriptions. This enables the model to learn a joint embedding space, allowing it to process and understand both text and images simultaneously and more effectively. CLIP offers nine different models with different architectures:

Convolutional neural networks (CNNs):

- RN50;
- RN101;
- RN50×4;
- RN50×16;
- RN50×64.

Vision transformers (ViTs):

- ViT-B/32;
- ViT-B/16;
- ViT-L/14;
- ViT-L/14@336px.

**Figure 1:**
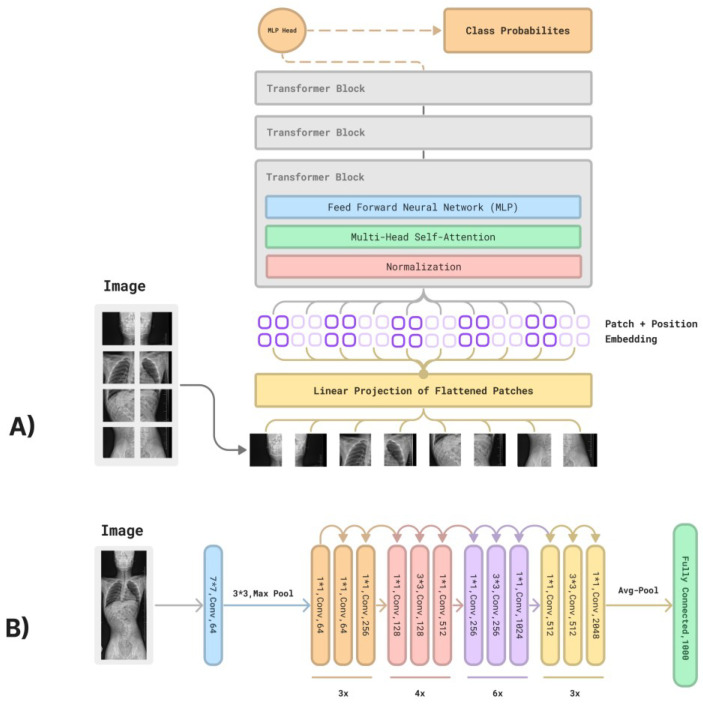
(**A**) A figure showing a simplified ViT (vision transformer) model. (**B**) A figure showing a simplified Rn50 model as an example of a convolutional neural network.

Although both architectures are powerful tools designed to understand the content found in images, they follow different approaches. In contrast to a traditional convolutional neural network, ViT is based on the transformer architecture that was primarily created for natural language processing tasks [4]. On the other hand, deep CNN ResNet models focus on local patterns or features (e.g., edges, textures), which afford them an advantage when processing matrix data such as images [5] (Figure 1).

Hyperparameters, which are variables that influence both the performance and subsequent operation of the model, are utilised to oversee the model’s learning process. Both convolutional neural networks (CNNs) and vision transformer networks (ViTs), which form integral parts of the CLIP model, have common hyperparameters (CH) in addition to hyperparameters that are uniquely dedicated to their respective architectures. Based on Radford’s research, in Table 1, we present a summary of the hyperparameters of the CLIP model [6].

The contrastive learning approach is the main reason behind CLIP’s success in being able to maximise the embeddings within an image and its textual description. At the same time, the similarity between image–text pairs that do not match is minimised. This objective allows the model to comprehend the semantics between images and text by learning a joint representation that encompasses their relationships and similarities [3].

The gold standard, for diagnostic purposes, to diagnose and assess the degree of scoliosis progression is a posturographic X-ray examination (stitching) that covers the entire spinal column. This examination is traditionally performed in two projections: anterior–posterior and lateral. This allows not only the degree of curvature to be determined but for the degree of rotation, the dynamics of the deformation process, or the planning the therapeutic procedure to be determined as well [7,8]. Scoliosis is a three-dimensional deformation of the spinal column where the curvature of the spine in the coronal plane exceeds 10 angular degrees as measured using the Cobb method [9].

Scoliosis classification is exceptionally extensive. It includes divisions based on the size of the curvature angle, age, aetiology, location, or number of curves. For the purposes of this work, we make the generalisation that scoliosis can be divided into the categories of single-curve (with a characteristic C-shape) and double-curve (in which the spine resembles the shape of the letter S, also known as S-shape) [10]. The current literature estimates the prevalence of adolescent idiopathic scoliosis (AIS) to be from 0.35% to 5.2%, with a screening study on a Turkish child population of 16,045 students finding that only 2.3% of the subjects were diagnosed with scoliosis (369 adolescents), with more children being diagnosed with single-curve scoliosis (69.3%) than double-curve scoliosis (29.3%) [11].

**Figure 2:**
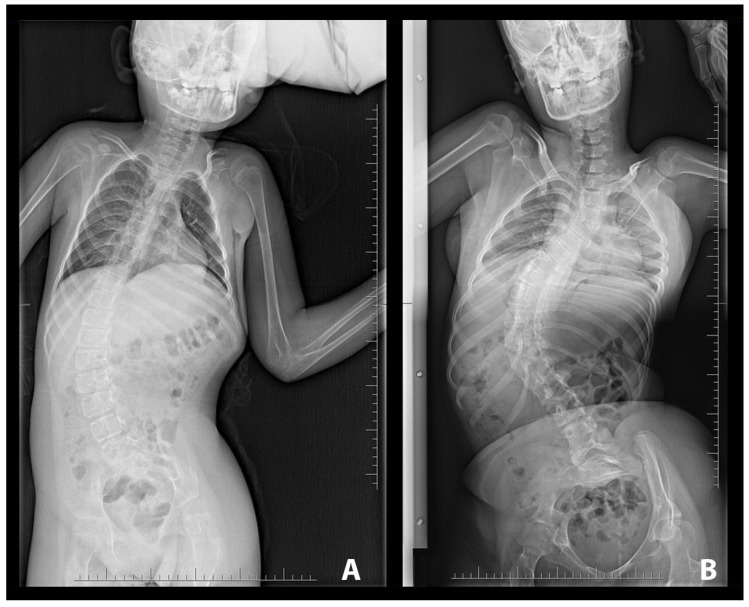
Example of single-curve scoliosis (C-shape). (**A**) Cobb angle measured between L4/L5 and Th9/Th8, equal to 45 degrees. (**B**) Cobb angle equal to 85 degrees measured between L2/L3 and Th6/Th5.

The degree of scoliosis severity is determined based on the size of the curvature angle as measured using the Cobb method. Severe scoliosis is described as spinal column curvature exceeding 40 degrees [12]. In the image below, we present examples of severe single-curve scoliosis (C-shape scoliosis) (Figure 2).

Using AI to analyse radiological images involves designing a complex and expensive system that is specifically trained for a dedicated medical problem, such as analysing radiological images to assess scoliosis [13]. Currently, OSAIMs are not trained for such challenges. However, considering the vast capabilities of CLIP, we decided to investigate and challenge the technology to analyse posturographic X-ray images of patients diagnosed with single-curve scoliosis. We chose to study severe cases of single-curve scoliosis because, in our opinion, the assessment of such advanced deformations is not a clinical challenge for radiologists or specialists dealing with spinal pathologies. We were only interested in determining the general evaluation of the deformation that CLIP could achieve by asking a series of questions, ranging from simpler questions to more complex ones, along with the presentation of X-ray images. The research hypotheses were defined as follows:

**H1.**  *All models implemented using the CLIP system will be able to detect scoliosis with a high level of sensitivity (sensitivity ≥ 80%).*

**H2.**  *All of the models implemented using the CLIP system will be able to detect single-curve scoliosis with a high level of sensitivity.*

Although CLIP and other OSAIM programs are not currently dedicated to medical image analysis, it is worth considering that the pace of technological development is so rapid that, in the future, they may become an alternative to expensive, complex AI models designed for specific medical problems.

## 2. Materials and Methods

This study was conducted at the Polish Mother’s Memorial Hospital Research Institute in Poland. The bioethics committee decided that the study of the obtained radiological images did not require committee approval. Posturographic images (stitching) in patients diagnosed with scoliosis in the anterior–posterior projection were collected from the years 2021 to 2022 (age range from 8 to 16 years old). To perform X-ray examination, medical indications are required. Consequently, we do not perform posturographic examinations on patients without spinal column deformations; therefore, the present study used images of patients who had previously been diagnosed with scoliosis. All of the images used in the study were anonymised. Consent was obtained from the patients’ legal guardians for the use of the X-ray images in the study. Inclusion criteria included technically correct images and those showing single-curve scoliosis with a degree of deformation above 40 degrees measured using the Cobb method. Image quality assessment involved checking for illegible images, incorrect image stitching, or framing. Exclusion criteria included scoliosis below or equal to 40 degrees of curvature, double-curve scoliosis, images not covering the entire spinal column, scoliosis after surgical correction with visible implants, or scoliosis with additional bone defects such as hyperkyphosis. From a database of 70 posturographic X-ray images, 23 posturographic images with visible severe single-curve scoliosis were included in the study. All examinations were performed using the same equipment. The X-ray images were not subjected to any modifications and were saved in JPEG format with a resolution of 2663 × 1277 px.

### 2.1. Manual Measurement

Analysis of the posturographic X-ray images was conducted independently by two neurosurgery specialists. RadiAnt software was used to evaluate the posturographic images and the Cobb angle measurements.

### 2.2. CLIP Methodology

The analysis was conducted using Python version 3.9 on Windows 11 (build 22621.1555) utilising the open-source codebase available on OpenAI’s official GitHub repository: https://github.com/openai/CLIP (accessed on 8 April 2023). The recognition quality of the images was investigated using 9 varieties of CLIP models, namely “RN50”, “RN101”, “RN50×4”, “RN50×16”, “RN50×64”, “ViT-B/32”, “ViT-B/16”, “ViT-L/14”, and “ViT-L/14@336px”. We used a code architecture consisting of a set of operations that apply an evaluation of a specified model against a set of images and text.

For each radiograph examined, the results were determined using four sets of questions (the statistical analysis only considered answers to the affirmative questions) (Table 2).

### 2.3. Statistical Analysis

Analyses were conducted using the R statistical language (version 4.1.1) on Windows 10 Pro 64 bit (build 19044) using the packages report (version 0.5.1.3) and caret (version 6.0.93).

The predictions that were obtained from the CLIP models in the form of probabilities ranging from 0 to 1 were compared with the actual data. For probabilities in the form of a real value, a cut-off value of 0.50 was used. Values equal to or higher than the cut-off value were treated as the presence of a feature (1.0) and as the absence of a feature otherwise (0.0).

To evaluate the quality of image recognition, true positives (*TP* (correctly indicated the presence of a condition)), false negatives (*FN* (incorrectly indicated the presence of a condition)), and the sensitivity were calculated based on Formula (1) (the inability to use other quality metrics (i.e., accuracy, specificity) was due to the fact that only images in which scoliosis was present were analysed (no radiographs of patients without scoliosis were examined)):

$$Sensitivity=\frac{TP}{TP+FN}$$

For each model, 5 sensitivity values were calculated (4 for individual question sets and 1 total value for all sets).

## 3. Results

A total of 23 posturographic X-ray images with visible severe single-curve scoliosis were included in this study. The minimum average degree of curvature was 42 degrees, while the highest average result was 88.5 degrees. The estimation results for predictive sensitivity are listed below (Table 3).

The following conclusions can be drawn from the data in Table 1: For the scoliosis question set, results with 100% sensitivity were obtained for seven of the nine CLIP models (except VitL14 and VitL14@336px). For the single-curve question set, high sensitivity levels were obtained for four of the nine models. None of the models were able to achieve a good level of image recognition for the C-shape question set. A result above 50% (the level indicating random guessing for dichotomous answers) was only achieved by VitB32 and RN50×64. The models that were tested were not suitable for detecting the Cobb angle (there were no correct answers obtained for this set of questions), and additional model training procedures are required. The highest score in terms of overall sensitivity for all question sets was obtained by the RN50 network. The lowest score in terms of overall sensitivity for all question sets was obtained by the RN101 network.

## 4. Discussion

This study’s results partially confirm our initial research hypothesis, showing that only seven out of nine tested CLIP system models are able to successfully recognise scoliosis using radiological images. Our second hypothesis was not confirmed because only four out of nine models responded to the single-curve question set successfully. Our assumptions regarding the accurate estimation of the curvature degree using AI were not confirmed. None of the selected models achieved high sensitivity when evaluating Cobb angles.

The observed phenomena can be attributed to the fact that the research on artificial intelligence that has been carried out in recent years has been extensively focused on addressing discrete medical challenges. This focus has necessitated the creation and deployment of intricate and precise algorithms that have been purpose-built for specific applications. These include, but are not limited to, Cobb angle analysis for assessing spinal deformities, breast cancer diagnosis, and providing support for patient monitoring in cardiovascular intensive care units [13,14,15].

Furthermore, it may be postulated that the performance efficacy of a given AI model is significantly contingent upon its architectural complexity. Simpler models may not deliver results that are as proficient as those obtained by their more complex counterparts, which analyse data in a more comprehensive manner. In the study conducted by Radford and his team, the performance of various available CLIP models was evaluated against multiple datasets, and zero-shot technology was benchmarked against a range of fully supervised classifiers. The findings of that research indicated that CLIP faces challenges when tasked with more intricate operations, such as while detecting tumours in lymph nodes, counting objects in synthetic scenes, and autonomous driving-related tasks such as recognising traffic signals [6].

Generally, vision transformers performed the best compared to the other models, especially ViT/L14 and ViT/L14@336px. They were able to analyse the image based on 14 × 14 patches, while their large-size (L) architecture is much more complex. These models have more layers, hidden units, and attention heads compared to the base-size (B) models, such as in the case of the other two ViT models. That said, why did these models perform the worst in identifying scoliosis? One reason could be the image size. ViT/L14@336px is able to adapt to images with a resolution of 336 × 336 px, but our posturographic X-ray images had a resolution of 2663 × 1277 px. Moreover, both ViT/L models divided the image into smaller patches, and their architecture forces multi-layered image analysis. X-ray images are rather static, less dynamic, and uniform units of information compared to, for example, images of cities or animals. In the case of ViT/L models, an analysis that is too detailed could negatively affect the final classification results for scoliosis compared to a ViT/B architecture, and models with this architecture type performed much better in this task. An additional argument could be the fact that the posturographic images that were generated originally had a black background, occupying 75% of the entire image surface. Due to the behaviour of visual transformers and dividing the input image into smaller parts, such a large amount of insignificant information in the picture could lead to incorrect results. At this point, it should be noted that further research is needed to analyse the potential of these two models, where normalised and prepared images without unnecessary backgrounds should be evaluated.

In the case of convolutional neural network (CNN) models, all of the studied models demonstrated a high sensitivity level in identifying scoliosis. This could be attributed to several of the features that are inherent to CNNs that make them particularly suited for the analysis of posturographic images.

Firstly, CNNs place strong emphasis on local features and patterns within images through the use of convolutions. Spinal X-ray images often contain numerous such features, including the shape of the vertebrae, their arrangement, and so on, which are critical for the detection of scoliosis.

Secondly, thanks to the integration of convolution and pooling layers, these networks exhibit enhanced flexibility when it comes to analysing images of varying resolutions.

Lastly, models such as ResNet, which possess a fixed depth and a more regular architectural structure compared to ViT, can have a positive impact on the classification process. The standardisation of this architecture type could result in improved accuracy and could ensure that these models are less susceptible to overfitting, further improving the robustness of scoliosis detection.

An interesting issue is also the discrepancy observed in the results when asking about the shape and type of scoliosis: whether it was single-curve or C-shape scoliosis. The term single-curve seems to be used much more often in the literature in relation to spinal deformities than C-shape. Therefore, the results show that only four out of the nine models achieved high sensitivity levels for questions related to single-curve scoliosis, and none were able to respond accurately to questions regarding C-shape scoliosis.

The absence of success in assessment of the Cobb angle by any of the models can be attributed to the fact that this evaluation, taking measurements using the Cobb method, is a highly specialised task and a fundamental skill for experts addressing spinal deformities. For general models such as CLIP, such a specialised challenge could be too sophisticated, which is why purpose-trained neural network models are typically employed for these kinds of tasks.

This begs the question, what led to the superior performance of the RN50 model overall compared to the other models? It is important to note that having more layers and a more complex model is not always synonymous with achieving better and more precise results. For instance, X-ray images, in contrast to more complex image types such as microscopic images, are considerably more simple. These images have a limited colour range, hence making them less intricate.

Mammography images, for example, demand a more rigorous analysis of different pixel intensities and their correlations. The RN101 model, despite having twice as many layers as the RN50 model, performed less efficiently in the overall assessment. This suggests that a higher number of layers may not always enhance the analysis but could potentially complicate it, thereby serving as a disadvantage.

At present, there are few studies that examine the potential of CLIP in medicine; however, in the table below, we present a summary of the most important studies that have used the CLIP architecture in biological research.

In Wang’s study, image features were extracted from functional magnetic resonance using CLIP, which encodes visual concepts with supervision from natural language captions. They then used voxelwise encoding models based on CLIP features to predict brain responses to real-world images from the Natural Scenes Dataset. The results show that CLIP explains up to 78% of variance in stimulus-evoked responses from individual voxels in the held-out test data and captures both global and fine-grained semantic dimensions represented within the visual cortex. The authors suggested that incorporating natural language into vision models can improve the prediction and understanding of higher visual cortex, and that human understanding of their environment forms an important dimension of visual representation [16].

In another study by Luo, the authors focused on using the CLIP model to design peptides that can bind to and degrade pathogenic proteins. The authors adapted recent models for contrastive language–image pretraining to devise a unified, sequence-based framework to design target-specific peptides. They also created a streamlined inference pipeline, termed Cut&CLIP, that efficiently selects peptides for downstream screening by leveraging known experimental binding proteins as scaffolds. The authors reported that their Cut&CLIP pipeline is able to efficiently select peptides for downstream screening by leveraging known experimental binding proteins as scaffolds [17].

However, in addition to CLIP technology, there are other models that have the ability to analyse images and their corresponding descriptions, such as LXMERT, ALIGN, and VILBERT. All of these models are based on the transformer architecture, but they employ different learning methods [4]. CLIP uses a contrastive learning model, while LXMERT, ALIGN, and VILBERT utilise various multimodal learning techniques, such as masked image and text prediction tasks [18,19]. This often affects performance and the applicability of each model to specific problems or tasks. Moreover, it is important to consider the generalisation ability of these models across different tasks, which has a significant impact on the choice of databases to be investigated. CLIP and ALIGN perform well on multitopic tasks [6,20], while LXMERT and VILBERT achieve strong results in more specialised ones [18,19]. As shown, there is no ideal model that can analyse every possible problem due to the complexity of the data.

Considering their architecture, it might seem that models such as LXMERT or VILBERT would be much better at handling the problem of evaluating medical images. Unfortunately, due to the lack of information and in-depth research, assessing their capabilities has instead become the basis for further experiments. It would be worthwhile to compare CLIP’s capabilities against other OSAIMs. Although CLIP is not dedicated to medical image analysis, it has demonstrated a basic ability to evaluate posturographic X-ray images. It is worth considering the possibility of creating dedicated models such OSAIMs that are exclusively for the analysis of medical data, including data from radiological images.

In contrast, improving the performance and accuracy of the CLIP model from OpenAI for medical image analysis requires a multipronged approach. Firstly, the model should be fine-tuned using a specific dataset related to medical imaging. This process will enable the model to obtain detailed knowledge about the medical imaging domain, which will subsequently enhance its performance. Secondly, data augmentation techniques should be utilised. Techniques such as image transformation are capable of introducing necessary variations into the dataset, ultimately aiding the model in learning more invariant features and thus improving its robustness. Thirdly, any class imbalance issue present in medical datasets should be addressed. This can be achieved by employing methods such as oversampling, undersampling, or the synthetic minority oversampling technique, which will help the model to effectively learn from minority classes. In addition to techniques, multimodal learning should be incorporated into the model’s training process. By using both text and image data related to the medical domain, such as radiology reports, the model’s understanding and prediction accuracy can be significantly improved.

This study has certain limitations. First, this study had a poor database for AI model evaluation. Due to the low prevalence of scoliosis in children, intercentre studies from different countries are required. Furthermore, the main exclusion criterion, which was a technically incorrect examination, significantly reduced the size of the database. Another limitation was the number and detail of the questions asked. It is possible that more elaborate sets of questions would have contributed to CLIP having a better understanding of the operation. It would also be appropriate to extend the study to other AI models for a more in-depth analysis of OSAIMs.

## 5. Conclusions

The models using the CLIP artificial intelligence system that were selected for use in this study demonstrate the potential to perform preliminary evaluations of scoliosis using posturographic X-ray imagery. We posit that the adaptation of these AI models is essential to ensuring accurate analyses of medical images. Interestingly, the simpler CLIP models, which feature fewer layers, showed more effectiveness when working with the limited information inherent in posturographic X-ray images compared to more complex models that are tailored to handling intricate problems.

Equally significant is the necessity to refine our question-asking methodology, which currently requires improvement. Despite these challenges, it remains vital to acknowledge that the effective use of CLIP necessitates meticulous human guidance. This underscores the current limitation that general AI systems, though increasingly sophisticated, are not yet capable of autonomously conducting even basic image diagnostics without specialist involvement.

Researchers have highlighted the latent potential and development space within CLIP, which underscores the need for further research and exploration in the realm of open-source artificial intelligence models (OSAIMs). It is, however, noteworthy to consider that publicly accessible AI systems, such as ChatGPT, have the capacity to continuously improve around the clock at an efficiency level that is unattainable by humans. As a result, the learning curve and progression speed of AI programs surpass human capabilities, offering vast opportunities for future advancements in the field.

## Figures and Tables

**Table 1:** Presentation of common CLIP hyperparameters included at the bottom of the table, as well as CLIP-ResNet hyperparameters and CLIP-ViT hyperparameters.

	CLIP-ResNet Hyperparameters								
Model	Learning Rate	Embedding Dimension	Input Resolution	ResNet Blocks	ResNet Width	Text Transformer Layers	Text Transformer Width	Text Transformer Heads	
RN50	5 × 10^−4^	1024	224	(3, 4, 6, 3)	2048	12	512	8	
RN101	5 × 10^−4^	512	224	(3, 4, 23, 3)	2048	12	512	8	
RN50×4	5 × 10^−4^	640	288	(4, 6, 10, 6)	2560	12	640	10	
RN50×16	4 × 10^−4^	768	384	(6, 8, 18, 8)	3072	12	768	12	
RN50×64	3.6 × 10^−4^	1024	448	(3, 15, 36, 10)	4096	12	1024	16	
**CLIP-ViT Hyperparameters**									
**Model**	**Learning Rate**	**Embedding Dimension**	**Input Resolution**	**Vision Transformer Layers**	**Vision Transformer Width**	**Vision Transformer Heads**	**Text Transformer Layers**	**Text Transformer** **Width**	**Text Transformer Heads**
VitB16	5 × 10^−4^	512	224	12	768	12	12	512	8
VitB32	5 × 10^−4^	512	224	12	768	12	12	512	8
VitL14	4 × 10^−4^	768	224	24	1024	16	12	768	12
VitL14@336px	2 × 10^−5^	768	336	24	1024	16	12	768	12
**Common CLIP Hyperparameters**									
Hyperparameter					Value				
Batch size					32,768				
Vocabulary size					49,408				
Training epochs					32				
Maximum temperature					100.0				
Weight decay					0.2				
Warm-up iterations					2000				
Adam β1					0.9				
Adam β2					0.999 (ResNet), 0.98 (ViT)				
Adam epsilon					10−8 (ResNet), 10−6 (ViT)				

**Table 2:** The set of questions used for model evaluation.

Area of Interests	Questions
Scoliosis/no scoliosis (further—scoliosis)	“Is there any scoliosis on the radiograph?” “Is there no scoliosis on the radiograph?”
C-shape scoliosis/noC-shape scoliosis (further—C-shape)	“Is there a C-shape scoliosis on the radiograph?” “Is there no scoliosis C-shape on the radiograph?”
Single curve/non-single curve (further—single-curve)	“Is there a single curve scoliosis on the radiograph?” “Is there no single curve scoliosis on the radiograph?”
Cobb angle	“Cobb angle is 0–10 Degrees?” “Cobb angle is 11–20 Degrees?” “Cobb angle is 21–30 Degrees?” “Cobb angle is 31–40 Degrees?” “Cobb angle is 41–50 Degrees?” “Cobb angle is 51–60 Degrees?” “Cobb angle is 61–70 Degrees?” “Cobb angle is 71–80 Degrees?” “Cobb angle is above 81 Degrees?”

**Table 3:** Prediction results for the individual models for both the individual sets of questions and for the overall result.

Neural Network	Set of Questions	*n*	*TP*s	*FN*s	Sensitivity
RN50	Scoliosis	23	23	0	100%
	C-shape	23	0	23	0%
	Single-curve	23	23	0	100%
	Cobb angle	23	0	23	0
	**Overall**	**92**	**46**	**46**	**50.0%**
RN101	Scoliosis	23	23	0	100%
	C-shape	23	0	23	0%
	Single-curve	23	0	23	0%
	Cobb angle	23	0	23	0%
	**Overall**	**92**	**23**	**69**	**25.0%**
RN50×4	Scoliosis	23	23	0	100%
	C-shape	23	0	23	0%
	Single-curve	23	2	21	8.7%
	Cobb angle	23	0	23	0%
	**Overall**	**92**	**25**	**67**	**37.3%**
RN50×16	Scoliosis	23	23	0	100%
	S-shape	23	0	23	0%
	Single-curve	23	1	22	4.3%
	Cobb angle	23	0	23	0%
	**Overall**	**92**	**24**	**68**	**26.1%**
RN50×64	Scoliosis	23	23	0	100%
	C-shape	23	14	9	60.9%
	Single-curve	23	3	20	13.0%
	Cobb angle	23	0	23	0%
	**Overall**	**92**	**40**	**52**	**43.5%**
VitB16	Scoliosis	23	23	0	100%
	C-shape	23	0	23	0%
	Single-curve	23	5	18	21.7%
	Cobb angle	23	0	23	0%
	**Overall**	**92**	**28**	**64**	**28.9%**
VitB32	Scoliosis	23	23	0	100%
	C-shape	23	15	8	65.2%
	Single-curve	23	0	23	0%
	Cobb angle	23	0	23	0%
	**Overall**	**92**	**38**	**54**	**41.3%**
VitL14	Scoliosis	23	2	21	9.5%
	C-shape	23	2	21	9.5%
	Single-curve	23	23	0	100%
	Cobb angle	23	0	23	0%
	**Overall**	**92**	**27**	**65**	**29.3%**
VitL14@336px	Scoliosis	23	1	22	4.3%
	C-shape	23	6	17	26.1%
	Single-curve	23	23	0	100%
	Cobb angle	23	0	23	0%
	**Overall**	**92**	**30**	**62**	**32.6%**

Note: *n*—group sample, *TP*s—true positives; *FN*s—false negatives.

## Data Availability

Not applicable.
